# Intrathecal drug delivery systems: A case series advancing surgical, clinical, and technological safety with broader implications for invasive neuromodulation therapies

**DOI:** 10.1177/2050313X251338563

**Published:** 2025-04-29

**Authors:** Bi Mo, Sandra Sacks, Jerry Markar

**Affiliations:** 1 Division of Pain Medicine, Department of Anesthesiology and Perioperative Medicine, David Geffen School of Medicine, University of California, Los Angeles, CA, USA; 2 Division of Hematology–Oncology, Department of Internal Medicine, David Geffen School of Medicine, University of California, Los Angeles, CA, USA

**Keywords:** intrathecal drug delivery systems, neuromodulation, neurological complications, surgical competence, cybersecurity

## Abstract

Intrathecal drug delivery systems (IDDS) represent an advanced modality of invasive pharmacological neuromodulation, providing efficacious treatment for terminal malignant pain as well as select chronic noncancer pain conditions. Although intrathecal drug delivery systems offer the potential for reduced systemic adverse effects compared to conventional routes, they are not without significant complications, including infections, device dysfunction, and severe neurological injuries. Moreover, the integration of network-based smart-device applications into intrathecal drug delivery system control interfaces introduces a concomitant elevation in risks associated with software errors and cybersecurity vulnerabilities. Case 1: a 77-year-old male receiving intrathecal methadone therapy, after the failure of first- and second-line agents, developed a catheter-tip spinal granuloma resulting in irreversible paraplegia secondary to thoracic spinal cord compression. Case 2: a 37-year-old female with a history of longstanding depression experienced a severe surgical site infection attributable to suboptimal surgical techniques during intrathecal drug delivery system implantation. This complication led to septic shock and meningitis, necessitating device removal and prolonged intravenous antibiotic therapy, though she ultimately recovered without permanent neurological deficits. Case 3: a 67-year-old female encountered acute opioid withdrawal and subsequent hospitalization as a consequence of a tablet-based interrogation platform’s software error in timekeeping that miscalculated her intrathecal drug delivery system refill date and recovered without enduring neurological sequelae. Invasive neuromodulation therapies, including intrathecal drug delivery systems, present multifaceted challenges that necessitate rigorous patient and therapeutic agent selection, meticulous risk factor mitigation, continuous neuromonitoring, and prompt detection of subtle neurological changes indicative of potential complications. This analysis delineates three critical domains: first, clinical vigilance and enhanced monitoring protocols are essential for the early identification of severe complications, such as granuloma formation; second, an educational paradigm shift, standardized, comprehensive surgical training in fellowship programs is required to ensure technical proficiency, optimal postoperative management, and an in-depth understanding of psychosocial factors; and third, technological leadership, the adoption of app-based management systems on consumer platforms introduces vulnerabilities including software malfunctions and cybersecurity threats, thereby necessitating that physicians advocate for stringent safety standards and robust regulatory oversight. Collectively, these strategies are indispensable for enhancing the safety and efficacy of invasive neuromodulation therapies and transforming the landscape of chronic pain management.

## Background

Intrathecal drug delivery systems (IDDS) are increasingly recognized as an effective neuromodulatory intervention for patients with terminal malignant pain and neuromuscular spasticity refractory to conventional oral dosing regimens.^1^ Their utility has been extended to certain cases of chronic nonmalignant pain, particularly in patients who are unresponsive to standard therapies or who experience significant systemic adverse effects from oral regimens.^1,2^ Despite these benefits, the application of IDDS in chronic nonmalignant pain remains contentious due to the protracted duration of therapy and the heightened risk of complications from both pharmacological and device-related origins.^2–4^ Currently, the FDA and European Medicines Agency have approved only morphine, baclofen, and Ziconotide for intrathecal administration.^3,5^

While intrathecal administration significantly reduces drug dosage compared to oral or parenteral routes, thus potentially mitigating systemic side effects and enhancing pharmacokinetic profiles, IDDS are nonetheless associated with considerable risks. These include central nervous system (CNS) and device/catheter site infections, mechanical device malfunctions, wound complications, granuloma formation, and subsequent severe neurological sequelae, such as motor and/or sensory deficits and paralysis.^3,4,6^

Moreover, a standardized consensus regarding neurological monitoring protocols and assessment of surgical competence is conspicuously lacking. This gap in standardization predisposes patients, particularly those with multifaceted comorbidities, to elevated morbidity and mortality risks. The convergence of IDDS operational software with consumer smart devices further complicates the landscape, introducing additional hazards related to software malfunctions and cybersecurity vulnerabilities that may precipitate targeted cyberattacks, thereby disrupting critical therapeutic interventions.^7^

In this series, we present three cases that underscore the necessity of mitigating harm associated with IDDS therapy. These cases highlight the critical importance of early detection of neurological changes in patients with complex comorbidities, the imperative of surgical competence and adherence to stringent perioperative antimicrobial protocols, and the evolving role of physician leadership in maintaining technological safeguards in an era of rapid innovation in invasive therapeutic modalities.

## Case presentations

### Case 1: Catheter granuloma and the imperative of neurological vigilance

A 77-year-old male with a medical history notable for type 2 diabetes mellitus, restrictive cardiomyopathy, and pulmonary hypertension, with complications including arrhythmia requiring chronic anticoagulation, was treated with intrathecal methadone (6 mg/day at a concentration of 25 mg/mL) for chronic low back and lower extremity pain secondary to lumbosacral failed back syndrome and peripheral neuropathy. His prior therapeutic course was complicated by intolerance to intrathecal trials with morphine (resulting in histamine release syndrome), hydromorphone (histamine release syndrome), fentanyl (severe pruritus), and Ziconotide (ataxia/dizziness). Following a successful methadone trial, performed by an operating surgeon not listed as an author, the patient received a permanent implant of an IDDS (SynchroMed II system; Medtronic, Minneapolis, MN, USA). Although initial pain relief was satisfactory, the patient subsequently developed significant neurological deterioration <2 years after the initiation of IDDS therapy.

He presented to our tertiary academic center with an American Spinal Injury Association Impairment Scale grade A deficit ~1 month following his most recent IDDS refill. His neurological examination revealed a complete loss of motor and sensory function from the T8 level downward. Notably, prior assessments performed before the refill and reprogramming procedure had not indicated any deviation from his baseline status.

**Figure 1.**
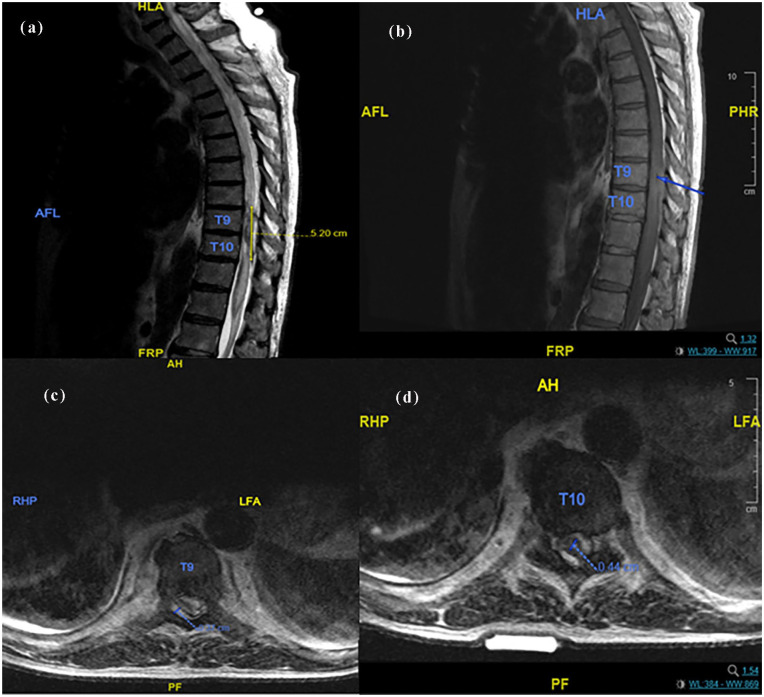
Thoracic MRI without contrast. (a) Sagittal. T2-weighted, demonstrating diffuse hyperintensity, suggestive of cord edema. (b) Sagittal. T1-weighted, indicating tip of the intrathecal catheter at T9 (blue arrow). (c) Axial. T2-weighted, showing right dorsal epidural fluid collection, measuring 0.37 cm, with mass effect. (d) Axial. T2-weighted at T10 level demonstrating heterogeneous collection suggestive of granuloma or hematoma measuring 0.44 cm.

During the interval preceding his paraplegic presentation, the patient experienced multiple admissions at external facilities for symptoms including progressive fatigue, lower extremity edema, and motor weakness, symptoms initially attributed to an exacerbation of his cardiopulmonary comorbidities. Despite the progressive nature of his lower extremity weakness, there was no documented investigation for potential IDDS-related complications. The patient’s clinical course culminated in profound neurological impairment characterized by bowel and urinary incontinence and complete loss of motor and sensory function below the mid-thoracic level. Emergent MRI studies revealed diffuse thoracic cord edema at the T9–T10 levels secondary to mass compression, raising suspicion for a drug-induced granuloma (Figure 1). Lumbar puncture for cerebrospinal fluid (CSF) analysis demonstrated a predominantly inflammatory response, although an infectious etiology could not be definitively excluded by the consulting Infectious Disease specialist. Following a multidisciplinary discussion with Neurosurgery and Neurology regarding the dismal prognosis, the patient and his family elected to transition to comfort care, and death was pronounced on hospital day 9. An autopsy was not performed in accordance with the family’s wishes.

### Case 2: Surgical competency and risk management in fellowship training

A 37-year-old female with a history of bipolar disorder and anxiety was referred for low back pain secondary to post-laminectomy syndrome after failing multiple epidural and spinal cord stimulation (SCS) therapies. Following a successful intrathecal opioid trial with hydromorphone, a Medtronic SynchroMed II pump was implanted by a surgical team not affiliated with this report.

Her initial postoperative course was unremarkable until postoperative day 9 when a follow-up evaluation revealed wound erythema at both surgical sites. These changes were initially attributed to a contact dermatitis reaction from the Opsite surgical dressing adhesive. In addition, a 2–0 Vicryl suture knot was noted protruding from the catheter insertion closure site and was trimmed by the evaluating fellow during her wound check. Given that the patient had completed a 7-day postoperative prophylactic antibiotic regimen and remained afebrile, no further antibiotics were prescribed, and the wound was managed conservatively via serial observation.

**Figure 2.**
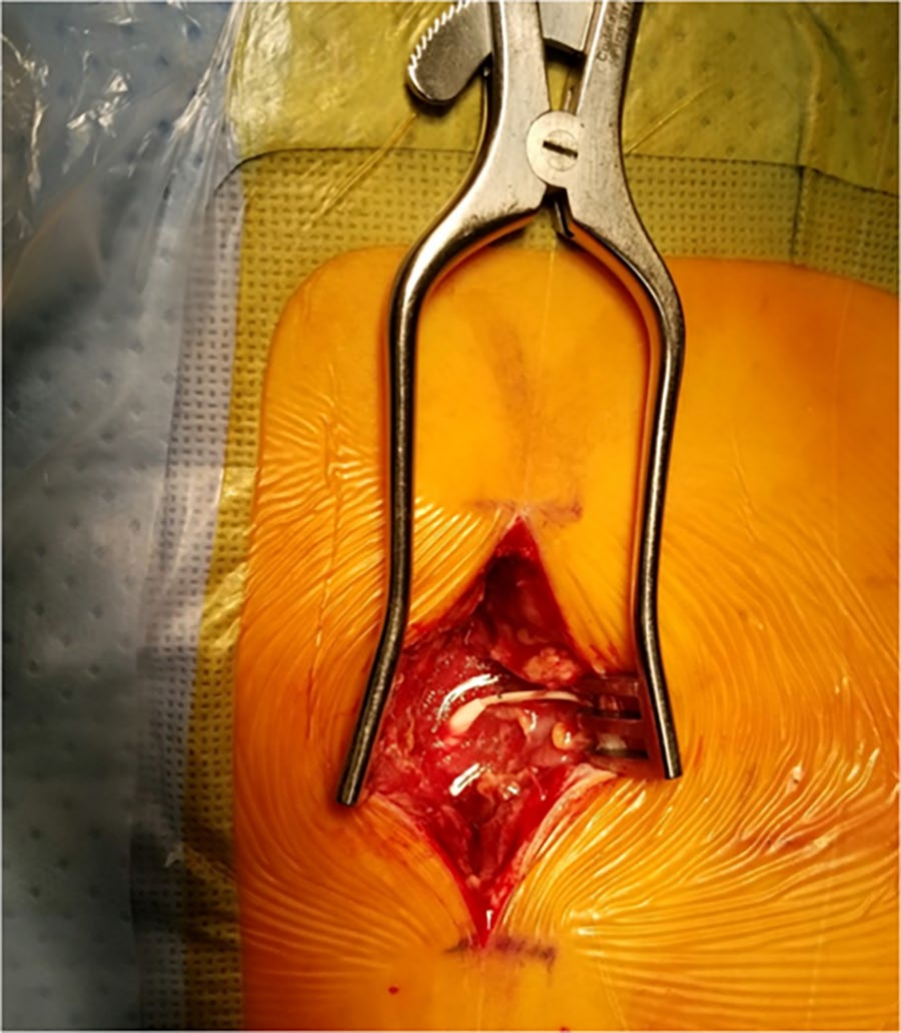
Intraoperative observation of purulent material at the lumbar spine catheter insertion site of the intrathecal drug delivery system.

**Figure 3.**
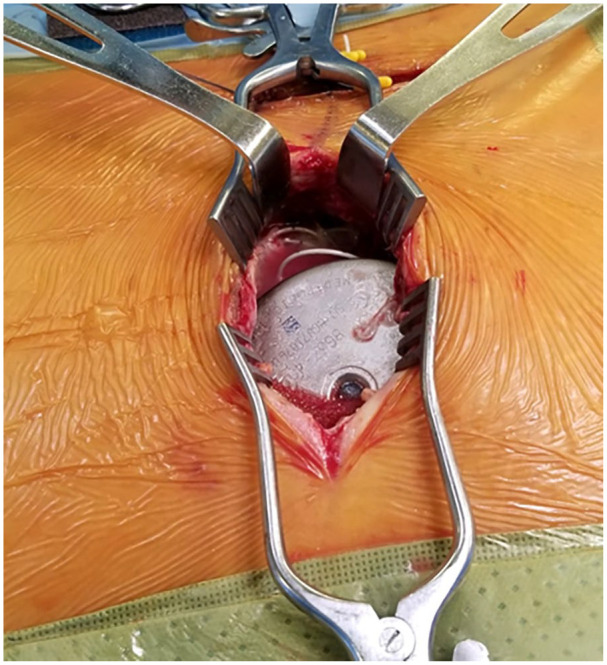
Intraoperative observation of purulent material at the lumbosacral spine intrathecal drug delivery system pump site.

Two weeks later, the patient presented to an external community hospital in septic shock with neurological signs consistent with acute bacterial meningitis, including photophobia, headache, and nuchal rigidity. She was subsequently transferred to our tertiary academic center for higher-level care. On examination, significant wound erythema and induration were noted at the catheter and pump incision sites, along with tenderness and warmth in the lumbar and lumbosacral regions. Neurological findings were indicative of meningitis with focal deficits, prompting emergent IDDS explantation, wound debridement/washout, and initiation of empirical intravenous vancomycin and ceftriaxone therapy following consultation with Infectious Disease. Intraoperative findings revealed copious purulent fluid at both the lumbar catheter insertion site (Figure 2) and the adjacent device pocket (Figure 3), with evidence of communication between the two sites via defects in the soft tissue layers. Intraoperative CSF cultures later confirmed *Staphylococcus epidermidis* as the pathogen.

The patient remained hospitalized for 2 weeks postoperatively until removal of the superficial retention suture and completion of a 6-week course of targeted intravenous vancomycin therapy via a peripherally inserted central catheter.

She ultimately recovered without permanent neurological sequelae, and 6 months postoperatively she resumed care with her primary pain specialist, receiving a subsequent IDDS implant 12 months later without perioperative complications.

### Case 3: Device security and physician leadership

**Figure 4.**
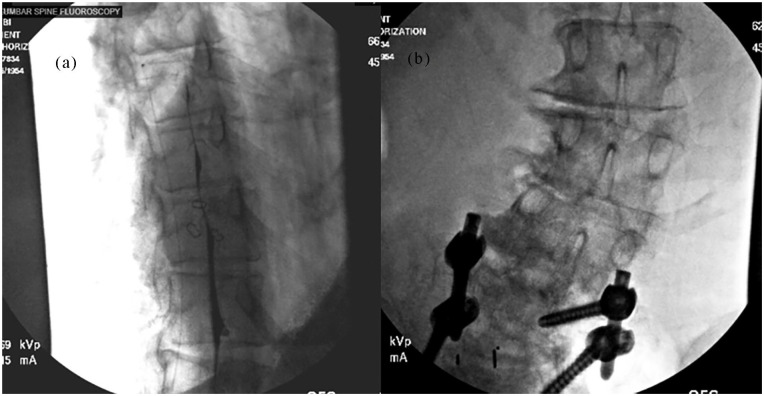
(a) Intrathecal drug delivery system myelogram. (b) Implanted catheter insertion site.

A 67-year-old female with chronic nonmalignant pain secondary to multilevel lumbar subluxation, degenerative disk disease, and idiopathic scoliosis was treated with a Medtronic SynchroMed II IDDS (Figure 4) after failing oral opioid regimens. The patient experienced significant complications due to an error in the network time protocol (NTP) of a tablet-based IDDS interrogation/reprogramming platform, which erroneously recalculated her refill date and precipitated acute opioid withdrawal, subsequent hospitalization, and increased patient risk.

In November of the preceding year, the patient underwent an IDDS refill at an outpatient ambulatory surgery center, with an anticipated refill date set for March of the following year. Approximately 4 weeks later, during an interval reprogramming session conducted with an alternative application-enabled Samsung consumer-grade tablet device, the system recalculated a new refill date ~1.5 months later in May, despite a 10% increase in the IDDS dosing. Multiple rechecks confirmed these findings, and the patient was discharged with an anticipated refill date in April, 2 weeks prior to the low reservoir volume alarm date in May.

In early March, the patient attended an interval evaluation and reported improved pain relief without acute concerns. However, ~1 week later, she was admitted to an external facility with exacerbated lower back pain and symptoms of opioid withdrawal, in addition to elevated transaminase levels concerning hepatitis secondary to venetoclax therapy for lymphocytic lymphoma. Urgent imaging, including axial spine MRIs, was negative for granuloma or infection. Retrospective review revealed that the patient’s IDDS alarm had been activated for several days prior to her initial presentation; however, both the patient and her spouse misinterpreted the alarm as originating from a household appliance, thus failing to alert her surgeon promptly.

The external facility, lacking the requisite expertise and equipment for IDDS management, was unable to refill or reprogram the device, resulting in an attempted oral substitution that proved intolerable due to nausea and suboptimal analgesia. Due to logistical and credentialing constraints, the patient’s device was not addressed until she was transferred to the tertiary academic center, where the initial implant was performed, 1 week later. A subsequent root cause analysis identified that the error was attributable to a battery discharge-induced internal clock NTP server connection failure in the clinic tablet.

The patient fully recovered from the acute opioid withdrawal episode without any neurological sequelae and subsequently resumed IDDS therapy, achieving sustained and consistent relief from her multifactorial lower back and leg pain during follow-up.

## Discussion

A retrospective analysis of 1001 FDA’s Manufacturer and User Facility Device Experience database reports involving SynchroMed (Medtronic) and Prometra (Flowonix, NJ, USA) by Goel et al. on IDDS reveals a broad spectrum of complications.^8^ Infection/erosion was the most frequently encountered issue, affecting 15.7% of cases, followed by pump motor stall (12.4%) and medication-related adverse events (11.8%). Catheter-specific complications were significant as well, including damage (8.2%), kinking (2.2%), migration (3.7%), and occlusion (0.5%), in addition to fluid collections around the catheter and pump (2.6%). Among pump-related issues, movement within the pocket occurred in 7.2% of reports, empty or low residual pump volume in 8.6%, high residual volume in 4.9%, and premature battery depletion in 2.4%. Biologic complications, though less common, encompassed granuloma (1.9%), spinal headache (1.8%), allergic reactions (0.6%), and pump site discomfort (6.8%), with spinal epidural hematomas reported in three cases and an overall mortality rate of 0.5%.^8^

These findings substantiate the significant risk of adverse events, including catastrophic outcomes, associated with IDDS. The inherently invasive nature of these permanently implantable neuromodulation modalities necessitates an intricate balance between therapeutic benefit and the risk of severe complications, such as irreversible neurological deficits and mortality.^2–6,8^

The devastating outcomes observed in complex patients who develop progressive and permanent neurological dysfunction underscore the critical need for heightened vigilance and the implementation of consensus-driven monitoring protocols for those at risk of neurological complications. This is especially pertinent in medical disciplines that may be less familiar with the nuances of spinal invasive therapies, including IDDS, dorsal root ganglion, and dorsal column stimulators. Notably, CNS infection risks in IDDS pose significant challenges owing to the necessity of repeatedly accessing the pump port for medication refills, device interrogations, and diagnostic imaging to assess catheter position and integrity.^8^ Moreover, any manipulation of the catheter can precipitate additional complications, such as hematoma formation, particularly in patients maintained on chronic therapeutic anticoagulation.^2,6^

Intrathecal granulomas, although rare (<3%), represent a severe complication of IDDS. They arise from localized inflammatory responses to intrathecal drug administration, with the highest risks correlated with morphine, the sole FDA-approved opioid therapy for IDDS.^3–6,9^ Granuloma-induced mass effects on the spinal cord and adjacent neural structures typically manifest as focal neurological deficits, a gradual or abrupt escalation of pain intensity without a clear inciting event, and an escalated requirement for higher doses of analgesic agents.^6^

Ziconotide, another FDA-approved agent for intrathecal pain therapy, is a synthetic CNS depressant composed of a 25-amino-acid polybasic peptide originally derived from the venom of the marine snail *Conus magus*. It is hydrophilic in nature and enables intrathecal trials to be performed via bolus dosing.^6^ Ziconotide selectively inhibits N-type voltage-gated calcium channels on Aδ and C-fiber pain afferents in the dorsal horn of the spinal cord, thereby reducing the release of neurotransmitters from pain-sensing neurons and modulating pain signaling.^9^ Notably, there have been no documented cases of granuloma formation associated with Ziconotide, and common adverse reactions associated with opioid formulations, such as respiratory depression, drug dependence, or withdrawal symptoms, are absent. Common neuropsychiatric side effects of Ziconotide include dizziness, confusion, nausea/vomiting, visual disturbances (e.g. amblyopia), vestibular disorders/ataxia, and encephalopathy.^9^ Unfortunately, in our case, the patient was not a candidate for Ziconotide due to previously reported ataxia and dizziness during the trial.

Methadone, a racemic mixture of D- and L-isomers, exhibits a dual pharmacologic profile: the D-isomer demonstrates minimal mu agonist activity coupled with noncompetitive NMDA receptor antagonism, while the L-isomer exerts both mu agonist and NMDA antagonist effects, thereby conferring analgesic properties.^10^ Limited data exist, though earlier non-randomized studies have reported the efficacy of intrathecal methadone in nonmalignant pain scenarios, such as in the perioperative management of total knee and hip arthroplasty patients, and for durations up to 6 months in chronic pain conditions.^10,11^ Comparatively, a more extensive body of clinical data supports the epidural use of methadone in malignancy-related pain, demonstrating significant pain reduction benefits without evidence of notable neurotoxicity or permanent neurological injury.^10,12,13^ Currently, there is no consensus or guideline endorsing the use of methadone in IDDS. The existing literature lacks robust clinical data supporting its safety and efficacy in this context. Consequently, comprehensive clinical studies are warranted to ensure patient safety, given the potential for severe neurological sequelae associated with intrathecal methadone administration.

A nuanced understanding of the pharmacokinetic properties of intrathecal agents is essential for optimizing therapeutic outcomes.^6^ Hydrophilic opioids, such as morphine and hydromorphone, exhibit extensive distribution within the CSF upon intrathecal administration, making them suitable for bolus dosing strategies.^6,14^ In contrast, lipophilic agents like fentanyl and bupivacaine are rapidly absorbed into epidural adipose tissue and systemic circulation.^14^ This pharmacokinetic profile necessitates continuous catheter infusions targeted at specific spinal segments to achieve effective analgesia in localized pain regions.^15^

The incorporation of bupivacaine, a non-opioid local anesthetic, as an adjuvant in intrathecal opioid therapy has been demonstrated to significantly attenuate opioid dosage escalation.^6,16^ This combination strategy is endorsed as first-line therapy by the Polyanalgesic Consensus Conference, given the limited evidence supporting alternative regimens.^6^ Notably, Ade et al. have demonstrated that intrathecal admixtures of bupivacaine with low-dose fentanyl or hydromorphone yield significant and sustained analgesia over extended follow-up periods, with no significant differences in efficacy observed between these combinations in a subset of the patient cohort.^15^ Furthermore, Veizi et al. demonstrated that low-dose strategies may offer only limited protection against granuloma formation when using hydromorphone, indicating that both the degree of drug exposure and the specific intrathecal agent are critical determinants in granuloma development.^17^ These findings advocate for the preferential use of safer therapeutic combinations and low-dose strategies, such as low-dose fentanyl with bupivacaine rather than hydromorphone, to reduce the risk of granuloma formation, a severe complication exemplified in Case 1.^15^ Moreover, these results underscore the critical need for further robust clinical data to comprehensively evaluate the efficacy and safety of methadone in the context of IDDS. Such evidence is imperative before its broader application in clinical practice, given the potential for detrimental effects; the off-label use of methadone should not be adopted without rigorous evaluation.

Nevertheless, irrespective of the intrathecal agent employed, maintaining a high index of clinical suspicion and performing prompt diagnostic evaluations is imperative for the effective identification of potential complications associated with the IDDS device or the pharmacologic agent. These complications include, but are not limited to, spinal catheter granulomas, infectious etiologies, or hematoma formation, all of which are paramount in reducing the likelihood of catastrophic outcomes such as permanent neurological damage, paralysis, or death.^2–6^

Unfortunately, the patient’s course of care was compromised by infrequent follow-up for IDDS refills over several months, coupled with a lack of proactive outreach from external medical teams to coordinate care with the implanting surgeon. This deficiency in clinical suspicion for IDDS-related complications significantly limited opportunities for early recognition and intervention, ultimately leading to substantial morbidity and subsequent mortality.

Furthermore, this case highlights the broader challenge of managing complex pain patients with significant comorbidities who are candidates for IDDS and other invasive neuromodulation therapies. In patients with conditions such as cardiopulmonary disease, subtle neurological symptoms may be erroneously attributed to an exacerbation of systemic illnesses rather than device-related complications. It is imperative that clinicians across specialties employ meticulous clinical examination and diagnostic testing, including a low threshold for imaging modalities, to definitively delineate underlying pathologies. By incorporating multidisciplinary care that involves interventional pain specialists, neurologists, and primary care physicians, a comprehensive approach can be established that ensures no aspect of patient care is overlooked. The development of such protocols and collaborative models is essential to mitigate the risk of delayed complication recognition.

It is, therefore, our view that heightened surveillance for changes in neurological function is paramount across all care settings for patients undergoing invasive neuromodulation therapies, particularly in those with complex comorbidities or evolving symptoms. Systematic neurological assessments, incorporating both imaging studies and symptom monitoring, are crucial to detect complications at an early, more treatable stage. The formulation and implementation of consensus guidelines for monitoring high-risk patients are needed to ensure that a heightened level of awareness and a recognized standard of care are maintained across primary, specialty, and long-term care teams, ultimately safeguarding overall patient outcomes with invasive implantable therapies.

The patient discussed in Case 2 further illustrates two critical considerations: the management of perioperative antimicrobial therapy and the necessity for enhanced surgical competency among trainees. The majority of infections associated with neuromodulation therapies are due to *Staphylococcus* species and are typically localized at the generator/pump pocket site, accounting for more than 50% of cases, whereas infection rates at the SCS electrode, catheter implantation site, and lumbar incision range from 8% to 17%.^18^ Notably, studies investigating postoperative antibiotic therapy across various surgical specialties, including cardiac, orthopedic, plastic, and spinal surgery, have not demonstrated a clear improvement in outcomes in the general population. Conversely, the practice of administering antibiotic therapy beyond 24 h postoperatively is prevalent in over 50% of cases, as reported by Deer et al.^18^ The Neurostimulation Appropriateness Consensus Committee consensus, in concurrence with the Surgical Care Improvement Project, recommends limiting and discontinuing antibiotics within 24 h postoperatively while considering extended coverage for high-risk patients.^18^ In the case under discussion, the patient had a prior incidence of superficial incision site infections during SCS therapy, which prompted an extension of antimicrobial therapy beyond the recommended 24-h period post-IDDS implantation. Despite this precaution, the patient still developed a significant deep tissue infection tracking to the CNS, resulting in meningitis that necessitated device explantation and targeted antimicrobial therapy secondary to suboptimal surgical techniques and delayed recognition of surgical site infection during the initial postoperative evaluation.

The imperativeness of a paradigm shift in fellowship training approaches is underscored by the growing integration of surgically invasive therapies into pain medicine. As invasive neuromodulation therapies, such as IDDS, become more prevalent, it is critical that pain fellows receive comprehensive surgical training to minimize the risk of neurological injury and infection in neuromodulation patients.^18–20^ Many trainees enter fellowship with little or no prior surgical experience, given that surgical internships are not compulsory in specialties such as Anesthesiology, Physical Medicine and Rehabilitation, Neurology, or Emergency Medicine. Combined with the inconsistency in IDDS implantation training experiences, wherein some fellows report zero cases during the fellowship, the current minimum case requirements appear insufficient to adequately prepare trainees for independent practice within the typical 12-month program duration.

An emphasis on surgical competency training in invasive neuromodulation therapies must encompass not only the acquisition of technical surgical skills but also a nuanced understanding of postoperative care, early complication identification, and appropriate management strategies. Complications such as incision and pocket site infections, although often preventable, require a deep comprehension of aseptic technique, meticulous wound care, and timely intervention to prevent progression to life-threatening conditions like septic shock.^18–20^ Standardizing fellowship curricula with clearly defined educational outcomes is essential to prepare future interventional pain specialists for the inherent complexities of neuromodulation therapies. Surgical training in pain medicine should be augmented by simulated practice, mentorship from experienced surgeons, interdisciplinary collaboration with departments such as Neurological Surgery, and direct involvement in a diverse array of cases to meet the required technical competency standards. Educational bodies, such as the ACGME, should consider the development of standardized competency assessments to ensure all fellows achieve a high level of proficiency before entering independent practice. Furthermore, research into surgical outcomes and complication rates among newly trained specialists may help identify areas for further curriculum development, thereby improving patient safety and quality of care.

Surgical competence extends beyond technical skills to encompass decision-making in patient selection, risk stratification, and postoperative follow-up. The case presented demonstrates the importance of recognizing potential high-risk features, such as a history of psychiatric comorbidities, including depression, which may predispose patients to poorer surgical and therapy outcomes, such as surgical site infections, due in part to an immunosuppressed state.^6,21,22^ It is postulated that depression and anxiety exacerbate the effects of physical or tissue injury postoperatively by precipitating an inflammatory cascade mediated by pro-inflammatory cytokines such as IL-1β, IL-6, and TNFα, ultimately impairing T-cell functionality and creating an immunocompromised state that heightens infection risk.^22^ Future interventional spine and pain specialists must be adept at integrating these multifactorial risks into their clinical decision-making to ensure that the benefits of IDDS and other neuromodulation therapies outweigh the potential hazards. Continued emphasis on patient selection criteria and the incorporation of formal education on perioperative risk management and optimization of psychosocial aspects of care will enable future specialists to maximize patient outcomes while minimizing complications.^6^

Beyond clinical challenges, advancements in IDDS technology and the increasing integration of invasive therapeutic controls into consumer-grade mobile platforms demand that medical professionals lead in device safety and cybersecurity in an era characterized by rapid technological evolution.^7^ This includes the integration of generative artificial intelligence (AI) in healthcare settings, which holds the potential to optimize patient care while concurrently introducing new vulnerabilities. The transition from proprietary hardware to app-based platforms for interrogating and programming neuromodulation therapies, including IDDS and SCS systems, introduces novel vulnerabilities, such as software-related failures, as exemplified in Case 3 when battery discharge interfered with the internal NTP. As the field of neuromodulation evolves, app-based technologies and remote telemetric capabilities to adjust therapy parameters in systems such as SCS and deep brain stimulation are becoming increasingly prevalent amid the burgeoning influence of AI technologies. While these advancements offer enhanced convenience and the ability to monitor and modify therapeutic response in real time, they also raise critical concerns regarding data security, device integrity, and overall reliability of patient care.^23,24^

Physicians must assume a leading role in the integration of these technologies into clinical practice. This leadership role encompasses advocating for stringent safety standards, contributing to the development of device and AI guidelines, and collaborating with industry stakeholders to proactively address emerging threats. Effective advocacy necessitates active engagement with regulatory bodies, technology developers, and healthcare institutions to ensure that patient safety remains the paramount priority amid ongoing innovation.^7,23,24^ Furthermore, fostering a culture of cybersecurity awareness within clinical teams, through ongoing education on device handling, timely software updates, and secure data practices, is vital to maintaining the integrity of neuromodulation therapies.^7,23,24^

The importance of physician leadership extends beyond ensuring technological safeguards; it also involves active participation in shaping the policy landscape that governs these advancements. By engaging in legislative initiatives and contributing to the formulation of industry standards, physicians can help establish a balanced framework that protects patient safety while simultaneously fostering innovation in an era of AI integration.^7^ Engaging in research to evaluate the impact of app-based neuromodulation management on clinical outcomes and developing best practices for its integration are critical steps that healthcare professionals must undertake. As app-based management systems and remote monitoring become more widespread, the onus is on the medical community to anticipate and address potential pitfalls while optimizing the benefits for patients.

## Conclusion

The evolution of invasive neuromodulation therapies, notably IDDS and SCS, represents a transformative advance in chronic pain management. This series highlights that while these therapies offer significant therapeutic benefits, they also introduce considerable clinical, technological, and educational challenges that must be addressed to optimize patient outcomes. Early detection of subtle neurological changes through rigorous, standardized monitoring remains essential for preventing irreversible deficits, and comprehensive surgical training, augmented by simulation-based education and standardized competency assessments, ensures that future practitioners are fully equipped to manage the complexities of IDDS implantation.

The cases presented herein underscore several critical issues: first, the risk of granuloma formation associated with intrathecal opioid use; second, the necessity for a nuanced approach to primary and adjuvant therapeutic selection and dosing strategies to minimize neurological sequelae; third, complications arising from extended postoperative antibiotic administration that deviate from current guidelines; and fourth, vulnerabilities inherent in consumer-grade device interfaces compounded by inadequate cybersecurity measures. Collectively, these observations advocate for a proactive reappraisal of device safety protocols and the integration of robust cybersecurity frameworks to mitigate software malfunctions and targeted cyberattacks.

Furthermore, the imperative for physician leadership is evident; clinicians must actively engage with interdisciplinary teams, regulatory bodies, and technology developers to shape policies that balance innovation with patient safety. Future research should concentrate on longitudinal studies that evaluate device performance, complication rates, and the efficacy of emerging cybersecurity measures. Ultimately, the confluence of advanced technological solutions, rigorous clinical protocols, and enhanced educational frameworks holds the promise of significantly improving the safety and efficacy of invasive neuromodulation therapies, thereby reducing procedure-related morbidity and mortality, and transforming the landscape of chronic pain management.
